# Supplementary material for: Complexome profiling of the Chlamydomonas psb28 mutant reveals TEF5 as an early PSII assembly factor
Source: Plant Cell. 2025 Mar 17;37(6):koaf055. doi: 10.1093/plcell/koaf055 (PMC12164590; doi:10.1093/plcell/koaf055)
Supplement: koaf055_Supplementary_Data [file koaf055_supplementary_data.zip › psb28_interactive.zip!/plots/intensity/qualityPlots/peptides/TEF14_Cre06.g256250.t1.2.html]

### Cre06.g256250.t1.2

Trivial name: TEF14  
SeqCoverage [median]: 27.00  
Mass: 20.57 kDa  
  
 

| Sequence | MissedCleavage | Mass [Da] | wtIntensity | muIntensity | Score |
| --- | --- | --- | --- | --- | --- |
| DFMASENLTDR | 0 | 1297.56 | 60361.50 | 112735.80 | 210.08 |
| DFMASENLTDRR | 1 | 1453.66 | 8525.92 | 16281.22 | 104.59 |
| GVTRDFMASENLTDR | 1 | 1710.80 | 3115.52 | 11791.98 | 153.24 |
| GVTRDFMASENLTDRR | 2 | 1866.90 | 411.60 | 1518.55 | 77.42 |
| LEQELVPVQK | 0 | 1181.67 | 9602.35 | 18918.51 | 205.83 |
| RLEQELVPVQK | 1 | 1337.77 | 63093.78 | 127094.76 | 158.56 |
| SGSQLESGDLK | 0 | 1119.54 | 48305.69 | 84630.52 | 261.35 |
| SVQAAAAK | 0 | 744.41 | 8669.13 | 44855.72 | 125.58 |
| SVQAAAAKGDAAGSK | 1 | 1330.68 | 7769.07 | 10668.14 | 89.87 |
| SVQAAAAKGDAAGSKR | 2 | 1486.79 | 6201.68 | 19148.62 | 100.45 |
|  |  |  | 216056.2 | 447643.8 |  |
